# Supplementary material for: Temporal TGF-β Supergene Family Signalling Cues Modulating Tissue Morphogenesis: Chondrogenesis within a Muscle Tissue Model?
Source: Int J Mol Sci. 2020 Jul 9;21(14):4863. doi: 10.3390/ijms21144863 (PMC7402331; doi:10.3390/ijms21144863)
Supplement: Supplementary file 1 [file ijms-21-04863-s001.pdf]

Table A. Comparisons of different genes expression between adjacent time points in each continuous stimulation group using two-way ANOVA.

Group	Time (day)	Genes				
		Col1α1	*Col2α1*	*ACAN*	*ALP*	*SOX9*
Control	7	0.17 ± 0.21	0.22 ± 0.10	0.88 ± 0.10	-0.79 ± 0.09	0.13 ± 0.07
	14	0.17 ± 0.19	-0.21 ± 0.14	0.98 ± 0.12	-1.06 ± 0.08	0.23 ± 0.01
	30	0.58 ± 0.17	-0.16 ± 0.08	1.31 ± 0.47	-1.30 ± 0.19	0.70 ± 0.09
	7 vs. 14 *P*-value	0.9987	**0.0001**	0.8527	**0.0145**	0.0513
	14 vs. 30 *P*-value	**0.014**	0.7822	0.2044	**0.0331**	**<0.0001**
rBMP-2	7	-0.66 ± 0.16	4.71 ± 0.12	3.91 ± 0.13	1.83 ± 0.06	0.72 ± 0.13
	14	-0.34 ± 0.19	0.67 ± 0.08	2.15 ± 0.18	-0.86 ± 0.11	0.40 ± 0.07
	30	0.23 ± 0.14	0.04 ± 0.75	2.04 ± 0.26	-1.12 ± 0.18	0.26 ± 0.05
	7 vs. 14 *P*-value	**0.025**	**<0.0001**	**<0.0001**	**<0.0001**	**0.0004**
	14 vs. 30 *P*-value	**0.0004**	0.1382	0.6802	**0.0194**	0.0725
rTGF-β_3	7	-0.37 ± 0.23	0.53 ± 0.47	1.98 ± 0.18	-0.84 ± 0.14	0.34 ± 0.15
	14	-0.38 ± 0.10	0.87 ± 0.06	2.50 ± 0.15	-0.92 ± 0.18	0.39 ± 0.06
	30	0.86 ± 0.07	NA	2.66 ± 0.20	-1.29 ± 0.17	0.36 ± 0.05
	7 vs. 14 *P*-value	0.9821	NA	**0.0016**	0.7578	0.6964
	14 vs. 30 *P*-value	**<0.0001**	NA	0.3406	**0.0085**	0.8434
rBMP-7	7	-0.62 ± 0.22	0.57 ± 0.09	1.57 ± 0.33	-0.78 ± 0.14	0.43 ± 0.04
	14	-0.01 ± 0.09	0.73 ± 0.16	2.78 ± 0.11	-0.92 ± 0.22	0.36 ± 0.05
	30	0.14 ± 0.11	0.95 ± 0.13	2.39 ± 0.11	-1.07 ± 0.17	0.32 ± 0.07
	7 vs. 14 *P*-value	**0.0001**	0.2433	**<0.0001**	NA	0.1404
	14 vs. 30 *P*-value	0.3004	0.1286	**0.0331**	NA	0.4651
rBMP-2 + rTGF-β_3	7	-0.60 ± 0.08	0.68 ± 0.03	2.49 ± 0.08	-0.77 ± 0.10	0.52 ± 0.07
	14	-0.43 ± 0.09	1.12 ± 0.18	2.17 ± 0.06	-1.04 ± 0.18	0.48 ± 0.05
	30	0.05 ± 0.10	0.09 ± 0.48	2.23 ± 0.10	-1.34 ± 0.05	0.31 ± 0.03
	7 vs. 14 *P*-value	**0.0219**	0.1023	**0.0001**	**0.0126**	0.4726
	14 vs. 30 *P*-value	**<0.0001**	**0.0022**	0.5367	**0.004**	**0.0005**
rBMP-2 + rBMP-7	7	-0.75 ± 0.08	0.67 ± 0.14	1.75 ± 0.23	-1.05 ± 0.21	0.40 ± 0.01
	14	-0.62 ± 0.20	0.82 ± 0.16	2.29 ± 0.17	-0.85 ± 0.19	0.36 ± 0.04
	30	-0.10 ± 0.01	NA	1.99 ± 0.17	-1.38 ± 0.09	0.28 ± 0.10
	7 vs. 14 *P*-value	0.2641	NA	**0.0018**	0.1791	0.5903
	14 vs. 30 *P*-value	**<0.0001**	NA	**0.0628**	**0.001**	0.1073
rTGF-β_3 + rBMP-7	7	-0.67 ± 0.11	0.73 ± 0.09	1.53 ± 0.21	-0.77 ± 0.14	0.37 ± 0.09
	14	-0.21 ± 0.16	0.69 ± 0.17	2.84 ± 0.24	-0.88 ± 0.05	0.51 ± 0.04
	30	0.47 ± 0.07	0.93 ± 0.20	2.37 ± 0.18	-0.81 ± 0.16	0.40 ± 0.03
	7 vs. 14 *P*-value	**0.0002**	0.931	**<0.0001**	0.3732	**0.01**
	14 vs. 30 *P*-value	**<0.0001**	0.1528	**0.0109**	0.7073	**0.0305**
rBMP-2 + rTGF-β_3 + rBMP-7	7	-0.80 ± 0.17	0.75 ± 0.12	2.49 ± 0.18	-0.49 ± 0.21	0.63 ± 0.17
	14	-0.25 ± 0.07	0.79 ± 0.49	2.25 ± 0.07	-0.64 ± 0.37	0.46 ± 0.13
	30	0.58 ± 0.10	0.84 ± 0.09	3.27 ± 0.13	-1.26 ± 0.13	0.49 ± 0.05
	7 vs. 14 *P*-value	**<0.0001**	0.9814	**0.0423**	0.6518	0.1334
	14 vs. 30 *P*-value	**<0.0001**	0.9764	**<0.0001**	**0.0061**	0.9563

All data were presented as mean of calibrated normalized relative quantity (CNRQ) ± standard deviation (SD). The comparations were performed using two-way ANOVA. The interaction between the stimulation duration and the culture sampling time in the rBMP-7 treated group was not significant when analysing the *ALP* expression. We defined $P<0.05$ as a statistically significant difference (in bold). *Col1α1*: *Collagen Type I Alpha 1*, *Col2α1*: *Collagen Type II Alpha 2*, *ACAN*: *Aggrecan*, *ALP*: *Alkaline phosphates*, *SOX9*: *SRY (Sex Determining Region Y)-Box 9*, NA: Not available.

Table B. Comparisons of different genes expression between adjacent time points in each single stimulation group using two-way ANOVA.

Group	Time (day)	Genes				
		Col1α1	*Col2α1*	*ACAN*	*ALP*	*SOX9*
Control	7	0.17 ± 0.21	0.22 ± 0.10	0.88 ± 0.10	-0.79 ± 0.09	0.13 ± 0.07
	14	0.17 ± 0.19	-0.21 ± 0.14	0.98 ± 0.12	-1.06 ± 0.08	0.23 ± 0.01
	30	0.58 ± 0.17	-0.16 ± 0.08	1.31 ± 0.47	-1.30 ± 0.19	0.70 ± 0.09
	7 vs. 14 *P*-value	0.9987	**0.0001**	0.8527	**0.0145**	0.0513
	14 vs. 30 *P*-value	**0.014**	0.7822	0.2044	**0.0331**	**<0.0001**
rBMP-2	7	-0.26±0.12	0.55±0.17	0.76±0.16	-0.85±0.12	0.10±0.10
	14	0.35±0.11	-0.21±0.19	1.08±0.61	-0.96±0.10	0.29±0.12
	30	0.67±0.15	-0.27±0.14	1.46±0.49	-1.04±0.36	-0.46±0.28
	7 vs. 14 *P*-value	**<0.0001**	**<0.0001**	0.3628	0.6808	0.0881
	14 vs. 30 *P*-value	**0.0021**	0.9829	0.2344	0.8876	**<0.0001**
rTGF-β3	7	0.20±0.15	0.58±0.26	0.43±0.10	-1.09±0.05	-0.02±0.16
	14	0.29±0.13	-0.50±0.20	0.44±0.23	-1.17±0.14	0.14±0.09
	30	0.72±0.10	-0.21±0.12	1.22±0.42	-1.33±0.07	-0.16±0.08
	7 vs. 14 *P*-value	0.6166	NA	0.9994	0.6855	**0.0395**
	14 vs. 30 *P*-value	**<0.0001**	NA	**<0.0001**	0.1673	**<0.0001**
rBMP-7	7	-0.08±0.12	0.53±0.18	1.14±0.39	-1.04±0.17	0.05±0.20
	14	0.31±0.09	-0.35±0.16	0.59±0.29	-0.98±0.21	0.09±0.03
	30	0.64±0.26	-0.22±0.15	1.61±0.28	-1.13±0.20	-0.41±0.13
	7 vs. 14 *P*-value	**0.0009**	**<0.0001**	**0.0044**	NA	0.8438
	14 vs. 30 *P*-value	**0.0041**	0.3794	**<0.0001**	NA	**<0.0001**
rBMP-2	7	-0.11±0.18	-0.22±0.22	1.23±0.40	-1.11±0.23	0.01±0.04
+	14	0.24±0.12	-0.42±0.01	0.03±0.10	-1.32±0.02	-0.04±0.08
rTGF-β3	30	0.60±0.18	-0.20±0.16	1.23±0.19	-1.15±0.10	-0.48±0.23
	7 vs. 14 *P*-value	**0.0001**	0.3083	**<0.0001**	**0.0378**	0.8091
	14 vs. 30 *P*-value	**0.0001**	0.213	**<0.0001**	0.1355	**<0.0001**
rBMP-2	7	-0.54±0.28	0.58±0.19	1.34±0.39	-1.23±0.11	-0.03±0.16
+	14	0.24±0.09	-0.36±0.34	0.45±0.40	-1.22±0.09	0.13±0.10
rBMP-7	30	0.73±0.17	0.25±0.08	1.17±0.19	-0.73±0.20	-0.34±0.08
	7 vs. 14 *P*-value	**<0.0001**	NA	**<0.0001**	0.9986	**0.0247**
	14 vs. 30 *P*-value	**<0.0001**	NA	**0.0002**	**<0.0001**	**<0.0001**
rTGF-β3	7	-0.15±0.09	0.33±0.27	0.71±0.52	-1.51±0.15	-0.33±0.06
+	14	0.33±0.31	-0.55±0.14	0.57±0.46	-1.26±0.12	0.28±0.31
rBMP-7	30	0.83±0.13	0.11±0.28	1.09±0.55	-0.80±0.15	-0.26±0.04
	7 vs. 14 *P*-value	**<0.0001**	**<0.0001**	0.9055	**0.0091**	**<0.0001**
	14 vs. 30 *P*-value	**<0.0001**	**<0.0001**	0.0914	**<0.0001**	**<0.0001**
rBMP-2	7	-1.14±0.54	0.14±0.24	0.82±0.60	-1.09±0.11	0.17±0.14
+	14	0.33±0.10	-0.24±0.32	0.91±0.53	-0.73±0.06	0.36±0.17
rTGF-β3	30	0.54±0.38	0.41±0.12	0.77±0.15	-0.52±0.15	-0.34±0.11
+	7 vs. 14 *P*-value	**<0.0001**	0.0634	0.9653	**0.0115**	0.0598
rBMP-7	14 vs. 30 *P*-value	0.5243	**0.0007**	0.8932	0.2308	**<0.0001**

All data were presented as mean of calibrated normalized relative quantity (CNRQ) ± standard deviation (SD). The comparations were performed using two-way ANOVA. The interaction between the stimulation duration and the culture sampling time in the rBMP-7 treated group was not significant when analysing the *ALP* expression. We defined $P<0.05$ as a statistically significant difference (in bold). *Col1α1*: *Collagen Type I Alpha 1*, *Col2α1*: *Collagen Type II Alpha 2*, *ACAN*: *Aggrecan*, *ALP*: *Alkaline phosphates*, *SOX9*: *SRY (Sex Determining Region Y)-Box 9*, NA: Not available.

Table C. The comparisons of *ACAN* expression between each experimental group with every other group using one-way ANOVA.

	**Adjusted P Value**					
	**Single Stimulation**			**Continuous Stimulation**		
	**7 Day**	**14 Day**	**30 Day**	**7 Day**	**14 Day**	**30 Day**
rBMP-2 vs. rTGF-β_3	0.8499	0.1859	0.9703	**<0.0001**	**0.0135**	**0.0037**
rBMP-2 vs. rBMP-7	0.7553	0.4744	0.9975	**<0.0001**	**<0.0001**	0.2648
rBMP-2 vs. rBMP-2+rTGF-β_3	0.5102	**0.003**	0.9733	**<0.0001**	>0.9999	0.8991
rBMP-2 vs. rBMP-2+rBMP-7	0.255	0.1933	0.9195	**<0.0001**	0.7834	>0.9999
rBMP-2 vs. rTGF-β_3+rBMP-7	>0.9999	0.4294	0.7672	**<0.0001**	**<0.0001**	0.3608
rBMP-2 vs. rBMP-2+rTGF-β_3+rBMP-7	>0.9999	0.9953	0.1038	**<0.0001**	0.9548	**<0.0001**
rTGF-β_3 vs. rBMP-7	0.0882	0.9989	0.7085	**0.0405**	0.0854	0.6021
rTGF-β_3 vs. rBMP-2+rTGF-β_3	**0.0358**	0.6753	>0.9999	**0.0056**	**0.0278**	0.0901
rTGF-β_3 vs. rBMP-2+rBMP-7	**0.0112**	>0.9999	>0.9999	0.5464	0.3564	**0.0014**
rTGF-β_3 vs. rTGF-β_3+rBMP-7	0.9265	0.9995	0.9991	**0.0172**	**0.0216**	0.4807
rTGF-β_3 vs. rBMP-2+rTGF-β_3+rBMP-7	0.7265	0.5674	0.5574	**0.0054**	0.1638	**0.0051**
rBMP-7 vs. rBMP-2+rTGF-β_3	>0.9999	0.3235	0.7202	**<0.0001**	**<0.0001**	0.9421
rBMP-7 vs. rBMP-2+rBMP-7	0.9876	0.9991	0.573	0.8476	**0.0002**	0.1353
rBMP-7 vs. rTGF-β_3+rBMP-7	0.632	>0.9999	0.3651	>0.9999	0.9988	>0.9999
rBMP-7 vs. rBMP-2+rTGF-β_3+rBMP-7	0.8716	0.8895	**0.0225**	**<0.0001**	**<0.0001**	**<0.0001**
rBMP-2+rTGF-β_3 vs. rBMP-2+rBMP-7	0.9997	0.6623	>0.9999	**<0.0001**	0.9117	0.723
rBMP-2+rTGF-β_3 vs. rTGF-β_3+rBMP-7	0.3879	0.3629	0.9989	**<0.0001**	**<0.0001**	0.9782
rBMP-2+rTGF-β_3 vs. rBMP-2+rTGF-β_3+rBMP-7	0.6583	**0.0205**	0.5449	>0.9999	0.992	**<0.0001**
rBMP-2+rBMP-7 vs. rTGF-β_3+rBMP-7	0.1761	0.9996	>0.9999	0.6531	**<0.0001**	0.1963
rBMP-2+rBMP-7 vs. rBMP-2+rTGF-β_3	0.3716	0.5807	0.6937	**<0.0001**	0.9998	**<0.0001**
rTGF-β_3+rBMP-7 vs. rBMP-2+rTGF-β_3+rBMP-7	0.9998	0.8589	0.8738	**<0.0001**	**<0.0001**	**<0.0001**

The comparations were performed by one-way ANOVA. We defined $P<0.05$ as a statistically significant difference (in bold). *ACAN*: *Aggrecan*.

Table D. The comparisons of *SOX9* expression between each experimental group with every other group using one-way ANOVA.

	**Adjusted P Value**					
	**Single Stimulation**			**Continuous Stimulation**		
	**7 Day**	**14 Day**	**30 Day**	**7 Day**	**14 Day**	**30 Day**
rBMP-2 vs. rTGF-β_3	0.8157	0.7043	0.0942	**<0.0001**	>0.9999	0.7148
rBMP-2 vs. rBMP-7	0.9987	0.3879	0.9998	**0.0021**	0.9622	0.971
rBMP-2 vs. rBMP-2+rTGF-β_3	0.9557	**0.0157**	>0.9999	0.0761	0.5506	0.9832
rBMP-2 vs. rBMP-2+rBMP-7	0.8004	0.6325	0.9286	**0.0006**	0.9655	>0.9999
rBMP-2 vs. rTGF-β_3+rBMP-7	**0.0002**	>0.9999	0.5442	**0.0002**	0.27	0.2975
rBMP-2 vs. rBMP-2+rTGF-β_3+rBMP-7	0.9881	0.995	0.9423	0.86	0.8293	**0.0074**
rTGF-β_3 vs. rBMP-7	0.9863	0.9995	0.2339	0.8482	0.9968	0.9977
rTGF-β_3 vs. rBMP-2+rTGF-β_3	>0.9999	0.4714	0.0525	0.1262	0.3351	0.9946
rTGF-β_3 vs. rBMP-2+rBMP-7	>0.9999	>0.9999	0.6467	0.9804	0.9973	0.831
rTGF-β_3 vs. rTGF-β_3+rBMP-7	**0.0125**	0.7885	0.9661	0.9996	0.1372	0.9964
rTGF-β_3 vs. rBMP-2+rTGF-β_3+rBMP-7	0.3108	0.2709	0.6149	**0.002**	0.619	0.301
rBMP-7 vs. rBMP-2+rTGF-β_3	0.9997	0.7853	0.9956	0.8452	0.092	>0.9999
rBMP-7 vs. rBMP-2+rBMP-7	0.9834	>0.9999	0.9954	0.9997	>0.9999	0.9926
rBMP-7 vs. rTGF-β_3+rBMP-7	**0.0012**	0.475	0.82	0.9823	**0.0293**	0.871
rBMP-7 vs. rBMP-2+rTGF-β_3+rBMP-7	0.8264	0.1015	0.997	0.0708	0.233	0.0856
rBMP-2+rTGF-β_3 vs. rBMP-2+rBMP-7	0.9999	0.5439	0.8255	0.5706	0.0956	0.9966
rBMP-2+rTGF-β_3 vs. rTGF-β_3+rBMP-7	**0.0045**	**0.0227**	0.3851	0.3109	0.9996	0.8285
rBMP-2+rTGF-β_3 vs. rBMP-2+rTGF-β_3+rBMP-7	0.5367	**0.0022**	0.8492	0.7023	0.9997	0.0696
rBMP-2+rBMP-7 vs. rTGF-β_3+rBMP-7	**0.0135**	0.7229	0.9952	0.9998	**0.0306**	0.4119
rBMP-2+rBMP-7 vs. rBMP-2+rTGF-β_3	0.2961	0.2212	>0.9999	**0.0231**	0.2406	**0.013**
rTGF-β_3+rBMP-7 vs. rBMP-2+rTGF-β_3+rBMP-7	**<0.0001**	0.9854	0.9928	**0.0074**	0.976	0.7191

The comparations were performed by one-way ANOVA. We defined $P<0.05$ as a statistically significant difference (in bold). *SOX9*: *SRY (Sex Determining Region Y)-Box 9*.

Table E. The comparisons of *Col2α1* expression between each experimental group with every other group using one-way ANOVA.

	Adjusted P Value					
	Single Stimulation			Continuous Stimulation		
	7 Day	14 Day	30 Day	7 Day	14 Day	30 Day
rBMP-2 vs. rTGF-β3	0.9979	0.3996	0.999	**<0.0001**	0.8896	NA
rBMP-2 vs. rBMP-7	>0.9999	0.965	0.9996	**<0.0001**	>0.9999	**0.0479**
rBMP-2 vs. rBMP-2+rTGF-β3	**<0.0001**	0.7782	0.9964	**<0.0001**	0.1112	>0.9999
rBMP-2 vs. rBMP-2+rBMP-7	>0.9999	0.9483	**0.0001**	**<0.0001**	0.9747	NA
rBMP-2 vs. rTGF-β3+rBMP-7	0.6923	0.2094	**0.01**	**<0.0001**	>0.9999	**0.0383**
rBMP-2 vs. rBMP-2+rTGF-β3+rBMP-7	**0.0417**	>0.9999	**<0.0001**	**<0.0001**	0.9948	0.1035
rTGF-β3 vs. rBMP-7	0.9899	0.946	>0.9999	>0.9999	0.9818	NA
rTGF-β3 vs. rBMP-2+rTGF-β3	**<0.0001**	0.9983	>0.9999	0.9414	0.7371	NA
rTGF-β3 vs. rBMP-2+rBMP-7	>0.9999	0.9633	**0.0007**	0.9513	>0.9999	NA
rTGF-β3 vs. rTGF-β3+rBMP-7	0.3168	>0.9999	**0.0415**	0.7834	0.925	NA
rTGF-β3 vs. rBMP-2+rTGF-β3+rBMP-7	**0.0094**	0.523	**<0.0001**	0.7182	0.999	NA
rBMP-7 vs. rBMP-2+rTGF-β3	**<0.0001**	0.9995	>0.9999	0.9783	0.2288	0.0667
rBMP-7 vs. rBMP-2+rBMP-7	0.9998	>0.9999	**0.0006**	0.9834	0.9988	NA
rBMP-7 vs. rTGF-β3+rBMP-7	0.7974	0.7915	**0.0343**	0.8629	>0.9999	>0.9999
rBMP-7 vs. rBMP-2+rTGF-β3+rBMP-7	0.0624	0.9897	**<0.0001**	0.8038	>0.9999	0.9978
rBMP-2+rTGF-β3 vs. rBMP-2+rBMP-7	**<0.0001**	0.9999	**0.0011**	>0.9999	0.5335	NA
rBMP-2+rTGF-β3 vs. rTGF-β3+rBMP-7	**0.0024**	0.9692	0.0561	0.9998	0.1363	0.0548
rBMP-2+rTGF-β3 vs. rBMP-2+rTGF-β3+rBMP-7	0.1173	0.876	**<0.0001**	0.9992	0.3963	0.1411
rBMP-2+rBMP-7 vs. rTGF-β3+rBMP-7	0.5421	0.8323	0.7893	0.9997	0.9866	NA
rBMP-2+rBMP-7 vs. rBMP-2+rTGF-β3	**0.0241**	0.9825	0.7486	0.9986	>0.9999	NA
rTGF-β3+rBMP-7 vs. rBMP-2+rTGF-β3+rBMP-7	0.6923	0.298	0.0656	>0.9999	0.998	0.9991

The comparations were performed by one-way ANOVA. We defined $P<0.05$ as a statistically significant difference (in bold). *Col2α1*: *Collagen Type II Alpha 1*, NA: not available.

Table F. The comparisons of *Col1α1* expression between each experimental group with every other group using one-way ANOVA.

	**Adjusted P Value**					
	**Single Stimulation**			**Continuous Stimulation**		
	**7 Day**	**14 Day**	**30 Day**	**7 Day**	**14 Day**	**30 Day**
rBMP-2 vs. rTGF-β_3	0.111	0.9994	>0.9999	0.1505	0.9995	<0.0001
rBMP-2 vs. rBMP-7	0.9383	>0.9999	>0.9999	>0.9999	**0.0228**	0.8853
rBMP-2 vs. rBMP-2+rTGF-β_3	0.9813	0.9653	0.9995	0.9994	0.9697	0.186
rBMP-2 vs. rBMP-2+rBMP-7	0.6306	0.9584	0.9998	0.9888	0.0661	**0.0009**
rBMP-2 vs. rTGF-β_3+rBMP-7	0.9963	>0.9999	0.9171	>0.9999	0.853	**0.0191**
rBMP-2 vs. rBMP-2+rTGF-β_3+rBMP-7	**0.0001**	>0.9999	0.9798	0.8765	0.97	**0.0003**
rTGF-β_3 vs. rBMP-7	0.6734	>0.9999	0.998	0.2944	**0.0061**	**<0.0001**
rTGF-β_3 vs. rBMP-2+rTGF-β_3	0.5263	0.9996	0.9824	0.378	0.9996	**<0.0001**
rTGF-β_3 vs. rBMP-2+rBMP-7	**0.0012**	0.9993	>0.9999	**0.0223**	0.1904	**<0.0001**
rTGF-β_3 vs. rTGF-β_3+rBMP-7	0.3927	>0.9999	0.9901	0.1217	0.5577	**<0.0001**
rTGF-β_3 vs. rBMP-2+rTGF-β_3+rBMP-7	**<0.0001**	>0.9999	0.8771	**0.0062**	0.7928	**0.0041**
rBMP-7 vs. rBMP-2+rTGF-β_3	>0.9999	0.9979	>0.9999	>0.9999	**0.0016**	0.8916
rBMP-7 vs. rBMP-2+rBMP-7	0.096	0.997	0.9972	0.9214	**<0.0001**	**0.0301**
rBMP-7 vs. rTGF-β_3+rBMP-7	0.9998	>0.9999	0.8206	0.9997	0.3951	**0.0005**
rBMP-7 vs. rBMP-2+rTGF-β_3+rBMP-7	<0.0001	>0.9999	0.9963	0.6816	0.2086	**<0.0001**
rBMP-2+rTGF-β_3 vs. rBMP-2+rBMP-7	0.1547	>0.9999	0.9779	0.8618	0.431	0.406
rBMP-2+rTGF-β_3 vs. rTGF-β_3+rBMP-7	>0.9999	0.9841	0.6608	0.9981	0.2739	**<0.0001**
rBMP-2+rTGF-β_3 vs. rBMP-2+rTGF-β_3+rBMP-7	**<0.0001**	0.9872	0.9999	0.5807	0.4879	**<0.0001**
rBMP-2+rBMP-7 vs. rTGF-β_3+rBMP-7	0.2337	0.9801	0.9925	0.9949	**0.0019**	**<0.0001**
rBMP-2+rBMP-7 vs. rBMP-2+rTGF-β_3	**0.0143**	0.9838	0.8614	0.9996	**0.0054**	**<0.0001**
rTGF-β_3+rBMP-7 vs. rBMP-2+rTGF-β_3+rBMP-7	**<0.0001**	>0.9999	0.3975	0.9151	>0.9999	0.7828

The comparations were performed by one-way ANOVA. We defined $P<0.05$ as a statistically significant difference (in bold). *Col1α1*: *Collagen Type I Alpha 1,* NA: not available.

Table G. The comparisons of *Col10α1* expression between each experimental group with every other group using one-way ANOVA.

	Adjusted P Value		
	Single Stimulation		
	**7 Day**	**14 Day**	**30 Day**
rBMP-2 vs. rTGF-β3	0.9808	0.9951	0.9997
rBMP-2 vs. rBMP-7	0.6843	0.1434	>0.9999
rBMP-2 vs. rBMP-2+rTGF-β3	0.7646	0.9984	0.9904
rBMP-2 vs. rBMP-2+rBMP-7	0.9797	0.5263	**0.0426**
rBMP-2 vs. rTGF-β3+rBMP-7	NA	>0.9999	0.9979
rBMP-2 vs. rBMP-2+rTGF-β3+rBMP-7	NA	0.9165	**0.0208**
rTGF-β3 vs. rBMP-7	0.9925	0.4868	0.9993
rTGF-β3 vs. rBMP-2+rTGF-β3	0.9977	>0.9999	0.8815
rTGF-β3 vs. rBMP-2+rBMP-7	>0.9999	0.9206	**0.0126**
rTGF-β3 vs. rTGF-β3+rBMP-7	NA	0.9644	>0.9999
rTGF-β3 vs. rBMP-2+rTGF-β3+rBMP-7	NA	0.9996	**0.0059**
rBMP-7 vs. rBMP-2+rTGF-β3	>0.9999	0.4126	0.9804
rBMP-7 vs. rBMP-2+rBMP-7	0.993	0.9919	**0.013**
rBMP-7 vs. rTGF-β3+rBMP-7	NA	0.0756	0.9955
rBMP-7 vs. rBMP-2+rTGF-β3+rBMP-7	NA	0.7896	**0.0054**
rBMP-2+rTGF-β3 vs. rBMP-2+rBMP-7	0.9979	0.8762	0.1568
rBMP-2+rTGF-β3 vs. rTGF-β3+rBMP-7	NA	0.9821	0.7995
rBMP-2+rTGF-β3 vs. rBMP-2+rTGF-β3+rBMP-7	NA	0.9984	0.0791
rBMP-2+rBMP-7 vs. rTGF-β3+rBMP-7	NA	0.3469	**0.0081**
rBMP-2+rBMP-7 vs. rBMP-2+rTGF-β3	NA	0.9956	>0.9999
rTGF-β3+rBMP-7 vs. rBMP-2+rTGF-β3+rBMP-7	NA	0.7838	**0.0037**

The comparations were performed by one-way ANOVA. We defined $P<0.05$ as a statistically significant difference (in bold). *Col10α1*: *Collagen Type X Alpha 1*, NA: not available.

Table H. The comparisons of *ALP* expression between each experimental group with every other group using one-way ANOVA.

	Adjusted P Value					
	Single Stimulation			Continuous Stimulation		
	**7 Day**	**14 Day**	**30 Day**	**7 Day**	**14 Day**	**30 Day**
rBMP-2 vs. rTGF-β_3	0.1704	0.1418	0.2968	**<0.0001**	0.9998	0.5789
rBMP-2 vs. rBMP-7	0.442	>0.9999	0.9951	**<0.0001**	0.9997	0.9995
rBMP-2 vs. rBMP-2+rTGF-β_3	0.1026	**0.001**	0.9763	**<0.0001**	0.8608	0.2588
rBMP-2 vs. rBMP-2+rBMP-7	**0.0036**	**0.035**	0.2417	**<0.0001**	>0.9999	0.1414
rBMP-2 vs. rTGF-β_3+rBMP-7	**<0.0001**	**0.0074**	0.536	**<0.0001**	>0.9999	0.0551
rBMP-2 vs. rBMP-2+rTGF-β_3+rBMP-7	0.1567	0.0659	**0.0044**	**<0.0001**	0.5944	0.7779
rTGF-β_3 vs. rBMP-7	0.999	0.2158	0.7354	0.9963	>0.9999	0.2854
rTGF-β_3 vs. rBMP-2+rTGF-β_3	>0.9999	0.5123	0.8546	0.9934	0.9784	0.9991
rTGF-β_3 vs. rBMP-2+rBMP-7	0.7452	0.9981	**0.0008**	0.3392	0.9988	0.985
rTGF-β_3 vs. rTGF-β_3+rBMP-7	**0.0008**	0.9121	**0.0034**	0.9892	>0.9999	**0.0004**
rTGF-β_3 vs. rBMP-2+rTGF-β_3+rBMP-7	>0.9999	**<0.0001**	**<0.0001**	**0.0101**	0.3371	>0.9999
rBMP-7 vs. rBMP-2+rTGF-β_3	0.9902	**0.0018**	>0.9999	>0.9999	0.9844	0.0967
rBMP-7 vs. rBMP-2+rBMP-7	0.3908	0.0586	0.0533	0.0902	0.998	**0.0468**
rBMP-7 vs. rTGF-β_3+rBMP-7	**0.0002**	**0.0131**	0.1675	>0.9999	>0.9999	0.1624
rBMP-7 vs. rBMP-2+rTGF-β_3+rBMP-7	0.9984	**0.0396**	**0.0006**	0.0574	0.3097	0.4654
rBMP-2+rTGF-β_3 vs. rBMP-2+rBMP-7	0.8714	0.8742	**0.031**	0.0776	0.7835	>0.9999
rBMP-2+rTGF-β_3 vs. rTGF-β_3+rBMP-7	**0.0017**	0.9953	0.1055	>0.9999	0.9072	**<0.0001**
rBMP-2+rTGF-β_3 vs. rBMP-2+rTGF-β_3+rBMP-7	>0.9999	**<0.0001**	**0.0003**	0.0671	0.0518	0.9847
rBMP-2+rBMP-7 vs. rTGF-β_3+rBMP-7	0.0555	0.9984	0.9993	0.0669	>0.9999	**<0.0001**
rBMP-2+rBMP-7 vs. rBMP-2+rTGF-β_3	0.7696	**<0.0001**	0.673	**<0.0001**	0.6932	0.9212
rTGF-β_3+rBMP-7 vs. rBMP-2+rTGF-β_3+rBMP-7	**0.001**	**<0.0001**	0.3454	0.0779	0.5189	**0.001**

The comparations were performed by one-way ANOVA. We defined $P<0.05$ as a statistically significant difference (in bold). *ALP*: *Alkaline phosphatase*.

Table I. The comparisons of positive area ratio (%) in Alcian blue staining over time in groups applied different morphogen(s) for only 48h or continuously using two-way ANOVA.

Group	Time (day)	Single Stimulation	Continuous Stimulation
rBMP-2	7	0.99 ± 0.14	6.20 ± 1.73
	14	0.70 ± 0.38	11.03 ± 3.27
	30	9.01 ± 1.45	23.31 ± 4.70
	7 vs. 14 *P*-value	0.9894	0.0863
	14 vs. 30 *P*-value	**0.0043**	**0.0002**
rTGF-β_3	7	1.30 ± 0.25	4.08 ± 1.10
	14	5.46 ± 1.21	4.55 ± 0.83
	30	4.41 ± 1.49	26.45 ± 2.36
	7 vs. 14 *P*-value	**0.0075**	0.9078
	14 vs. 30 *P*-value	0.6282	**<0.0001**
rBMP-7	7	5.29 ± 1.27	14.33 ± 5.76
	14	5.55 ± 1.03	33.22 ± 7.46
	30	6.41 ± 0.32	28.14 ± 3.63
	7 vs. 14 *P*-value	0.9968	**0.0003**
	14 vs. 30 *P*-value	0.9655	0.3305
rBMP-2	7	2.77 ± 0.34	4.44 ± 2.75
+	14	3.25 ± 0.31	12.60 ± 2.66
rTGF-β_3	30	8.59 ± 1.48	15.39 ± 4.35
	7 vs. 14 *P*-value	NA	NA
	14 vs. 30 *P*-value	NA	NA
rBMP-2	7	3.90 ± 1.04	10.08 ± 2.59
+	14	3.37 ± 0.89	36.12 ± 3.19
rBMP-7	30	0.65 ± 0.18	25.65 ± 3.79
	7 vs. 14 *P*-value	0.9578	**<0.0001**
	14 vs. 30 *P*-value	0.3656	**0.0004**
rTGF-β_3	7	7.81 ± 1.72	6.39 ± 0.56
+	14	2.17 ± 0.87	13.54 ± 2.19
rBMP-7	30	1.45 ± 0.20	26.28 ± 2.00
	7 vs. 14 *P*-value	**0.0013**	**0.0002**
	14 vs. 30 *P*-value	0.8233	**<0.0001**
rBMP-2	7	4.99 ± 0.48	6.24 ± 4.20
+	14	7.00 ± 1.51	17.00 ± 5.00
rTGF-β_3	30	2.47 ± 0.47	27.26 ± 2.08
+	7 vs. 14 *P*-value	0.6774	**0.0017**
rBMP-7	14 vs. 30 *P*-value	0.1732	**0.0024**

All data were presented as mean ± standard deviation (SD). The comparations were performed using two-way ANOVA. The interaction between the stimulation duration and the culture sampling time in the rBMP-2 + rTGF-β_3 treated group was not significant. We defined $P<0.05$ as a statistically significant difference (in bold). NA: not available.

Table J. The comparisons of positive area ratio (%) in alcian blue staining between each experimental group with every other group using one-way ANOVA.

	Adjusted P Value					
	Single Stimulation			Continuous Stimulation		
	**7 Day**	**14 Day**	**30 Day**	**7 Day**	**14 Day**	**30 Day**
rBMP-2 vs. rTGF-β3	0.9998	**0.0003**	**0.0004**	0.9839	0.4534	0.9216
rBMP-2 vs. rBMP-7	**0.0005**	**0.0002**	0.0543	0.0592	**<0.0001**	0.6034
rBMP-2 vs. rBMP-2+rTGF-β3	0.2849	0.0632	0.999	0.9946	0.9994	0.1113
rBMP-2 vs. rBMP-2+rBMP-7	**0.0177**	**0.0472**	**<0.0001**	0.7389	**<0.0001**	0.9827
rBMP-2 vs. rTGF-β3+rBMP-7	**<0.0001**	0.5547	**<0.0001**	>0.9999	0.9898	0.9398
rBMP-2 vs. rBMP-2+rTGF-β3+rBMP-7	**0.001**	**<0.0001**	**<0.0001**	>0.9999	0.5504	0.7941
rTGF-β3 vs. rBMP-7	**0.001**	>0.9999	0.2163	**0.011**	**<0.0001**	0.9974
rTGF-β3 vs. rBMP-2+rTGF-β3	0.499	0.1378	**0.001**	>0.9999	0.2217	**0.0117**
rTGF-β3 vs. rBMP-2+rBMP-7	**0.04**	0.1791	**0.0029**	0.2667	**<0.0001**	>0.9999
rTGF-β3 vs. rTGF-β3+rBMP-7	**<0.0001**	**0.0098**	**0.0224**	0.9741	0.1345	>0.9999
rTGF-β3 vs. rBMP-2+rTGF-β3+rBMP-7	**0.0022**	0.4943	0.2454	0.9822	**0.0168**	>0.9999
rBMP-7 vs. rBMP-2+rTGF-β3	0.0502	0.1124	0.1468	**0.0147**	**0.0001**	**0.0033**
rBMP-7 vs. rBMP-2+rBMP-7	0.5695	0.1473	**<0.0001**	0.6514	0.9768	0.9753
rBMP-7 vs. rTGF-β3+rBMP-7	**0.0495**	**0.0078**	**0.0002**	0.0687	**0.0002**	0.9954
rBMP-7 vs. rBMP-2+rTGF-β3+rBMP-7	0.9999	0.5628	**0.0018**	0.061	**0.0016**	>0.9999
rBMP-2+rTGF-β3 vs. rBMP-2+rBMP-7	0.7655	>0.9999	**<0.0001**	0.3322	**<0.0001**	**0.0211**
rBMP-2+rTGF-β3 vs. rTGF-β3+rBMP-7	**<0.0001**	0.8396	**<0.0001**	0.99	>0.9999	**0.0133**
rBMP-2+rTGF-β3 vs. rBMP-2+rTGF-β3+rBMP-7	0.1044	**0.0029**	**<0.0001**	0.9939	0.8338	**0.0064**
rBMP-2+rBMP-7 vs. rTGF-β3+rBMP-7	**0.0013**	0.7614	0.9584	0.782	**<0.0001**	>0.9999
rBMP-2+rBMP-7 vs. rBMP-2+rTGF-β3	0.7979	**0.004**	0.3081	0.7478	**0.0003**	0.9981
rTGF-β3+rBMP-7 vs. rBMP-2+rTGF-β3+rBMP-7	**0.023**	**0.0002**	0.8704	>0.9999	0.9428	>0.9999

The comparations were performed by one-way ANOVA. We defined $P<0.05$ as a statistically significant difference (in bold).

Table K. The comparisons of MOD in aggrecan immunohistochemistry over time in groups applied different morphogen(s) for only 48h or continuously using two-way ANOVA.

Group	Time (day)	Single Stimulation	Continuous Stimulation
rBMP-2	7	12.06 ± 3.31	3.40 ± 0.80
	14	2.68 ± 1.43	63.12 ± 33.30
	30	0.04 ± 0.01	24.02 ± 5.79
	7 vs. 14 *P*-value	0.6939	**0.0005**
	14 vs. 30 *P*-value	0.9706	**0.0124**
rTGF-β_3	7	10.72 ± 1.51	16.75 ± 6.85
	14	9.00 ± 3.82	25.37 ± 6.84
	30	2.58 ± 1.26	32.87 ± 15.08
	7 vs. 14 *P*-value	NA	NA
	14 vs. 30 *P*-value	NA	NA
rBMP-7	7	0.55 ± 0.06	23.77 ± 13.52
	14	3.33 ± 1.77	49.71 ± 28.26
	30	6.60 ± 4.49	45.23 ± 6.63
	7 vs. 14 *P*-value	NA	NA
	14 vs. 30 *P*-value	NA	NA
rBMP-2 + rTGF-β_3	7	7.24 ± 3.79	4.86 ± 2.73
	14	2.43 ± 1.45	45.64 ± 8.12
	30	17.23 ± 3.46	81.41 ± 42.04
	7 vs. 14 *P*-value	0.9406	**0.0375**
	14 vs. 30 *P*-value	0.5746	0.0692
rBMP-2 + rBMP-7	7	4.35 ± 2.21	30.56 ± 11.96
	14	3.32 ± 1.97	53.28 ± 9.60
	30	13.84 ± 1.80	21.65 ± 4.06
	7 vs. 14 *P*-value	0.9804	**0.0033**
	14 vs. 30 *P*-value	0.1693	**0.0002**
rTGF-β_3 + rBMP-7	7	1.99 ± 0.70	7.77 ± 2.05
	14	8.26 ± 1.89	19.67 ± 1.72
	30	5.19 ± 2.25	80.97 ± 21.23
	7 vs. 14 *P*-value	0.6687	0.2625
	14 vs. 30 *P*-value	0.906	**<0.0001**
rBMP-2 + rTGF-β_3 + rBMP-7	7	0.64 ± 0.36	5.20 ± 1.95
	14	4.53 ± 0.95	91.54 ± 18.52
	30	1.52 ± 0.91	44.44 ± 27.87
	7 vs. 14 *P*-value	NA	NA
	14 vs. 30 *P*-value	NA	NA

The target antigen in immunohistochemistry was aggrecan. All data were presented as mean ± standard deviation (SD). The comparations were performed using two-way ANOVA. The interaction between the stimulation duration and the culture sampling time in the rTGF-β_3, rBMP-7 and rBMP-2 + rTGF-β_3 + rBMP-7 treated group was not significant. We defined $P<0.05$ as a statistically significant difference (in bold). MOD: mean optical density, NA: not available.

Table L. The comparison of MOD of immunohistochemistry between each experimental group with every other group using one-way ANOVA.

	Adjusted P Value					
	Single Stimulation			Continuous Stimulation		
	**7 Day**	**14 Day**	**30 Day**	**7 Day**	**14 Day**	**30 Day**
rBMP-2 vs. rTGF-β_3	0.9945	0.9849	0.8729	0.3384	0.3204	0.9992
rBMP-2 vs. rBMP-7	**0.0002**	>0.9999	0.0516	**0.0426**	0.9877	0.8951
rBMP-2 vs. rBMP-2+rTGF-β_3	0.2125	>0.9999	<0.0001	>0.9999	0.949	0.051
rBMP-2 vs. rBMP-2+rBMP-7	**0.0116**	>0.9999	<0.0001	**0.0043**	0.9981	>0.9999
rBMP-2 vs. rTGF-β_3+rBMP-7	**0.001**	0.9927	0.1897	0.9931	0.1857	0.0536
rBMP-2 vs. rBMP-2+rTGF-β_3+rBMP-7	**0.0002**	>0.9999	0.9919	>0.9999	0.6409	0.9116
rTGF-β_3 vs. rBMP-7	**0.0009**	0.992	0.4477	0.9133	0.7847	0.9939
rTGF-β_3 vs. rBMP-2+rTGF-β_3	0.566	0.9813	<0.0001	0.4698	0.8975	0.133
rTGF-β_3 vs. rBMP-2+rBMP-7	**0.0473**	0.9919	**0.0005**	0.3017	0.6598	0.9966
rTGF-β_3 vs. rTGF-β_3+rBMP-7	**0.0039**	>0.9999	0.8564	0.7631	>0.9999	0.1391
rTGF-β_3 vs. rBMP-2+rTGF-β_3+rBMP-7	**0.0009**	0.9981	0.999	0.5042	**0.0135**	0.9959
rBMP-7 vs. rBMP-2+rTGF-β_3	**0.034**	>0.9999	**0.0008**	0.0682	>0.9999	0.4129
rBMP-7 vs. rBMP-2+rBMP-7	0.4645	>0.9999	**0.0264**	0.9258	>0.9999	0.8364
rBMP-7 vs. rTGF-β_3+rBMP-7	0.9914	0.9965	0.9942	0.1661	0.5807	0.427
rBMP-7 vs. rBMP-2+rTGF-β_3+rBMP-7	>0.9999	>0.9999	0.2031	0.0762	0.2184	>0.9999
rBMP-2+rTGF-β_3 vs. rBMP-2+rBMP-7	0.7519	>0.9999	0.6399	**0.0071**	0.9996	**0.039**
rBMP-2+rTGF-β_3 vs. rTGF-β_3+rBMP-7	0.1428	0.9905	**0.0002**	0.9994	0.7297	>0.9999
rBMP-2+rTGF-β_3 vs. rBMP-2+rTGF-β_3+rBMP-7	**0.0371**	>0.9999	<0.0001	>0.9999	0.1439	0.3881
rBMP-2+rBMP-7 vs. rTGF-β_3+rBMP-7	0.8891	0.9965	**0.0063**	**0.0189**	0.4517	**0.041**
rBMP-2+rBMP-7 vs. rBMP-2+rTGF-β_3	0.4905	>0.9999	**0.0002**	**0.0079**	0.3061	0.8576
rTGF-β_3+rBMP-7 vs. rBMP-2+rTGF-β_3+rBMP-7	0.9939	0.9994	0.5537	0.9998	**0.0068**	0.4017

The target antigen in the immunohistochemistry was aggrecan. The comparations were performed by one-way ANOVA. We defined $P<0.05$ as a statistically significant difference (in bold). MOD: mean optical density.
